# Active Wild Food Practices among Culturally Diverse Groups in the 21st Century across Latgale, Latvia

**DOI:** 10.3390/biology10060551

**Published:** 2021-06-18

**Authors:** Baiba Prūse, Andra Simanova, Ieva Mežaka, Raivo Kalle, Julia Prakofjewa, Inga Holsta, Signe Laizāne, Renata Sõukand

**Affiliations:** 1 Department of Environmental Sciences, Informatics and Statistics, Ca’ Foscari University of Venice, Via Torino 155, 30172 Venice, Italy; yuliya.prakofyeva@unive.it (J.P.); renata.soukand@unive.it (R.S.); 2 Institute for Environmental Solutions, “Lidlauks”, Priekuļi Parish, LV-4126 Priekuļi County, Latvia; ieva.mezaka@vri.lv (I.M.); indziniex@inbox.lv (I.H.); signe.laizane@vri.lv (S.L.); 3 Department of Latvian and Baltic Studies, Faculty of Humanities, University of Latvia, Rainis Boulevard 19, LV-1586 Riga, Latvia; 4 University of Gastronomic Sciences, Piazza Vittorio Emanuele 9, 12042 Pollenzo, Italy; raivo.kalle@mail.ee

**Keywords:** Latvia, local ecological knowledge, wild food plants, natural resources, foraging

## Abstract

**Simple Summary:**

A study in the bordering region of Latvia took place in order to investigate wild plant food uses. In total 72 interviewees reported food uses. The most represented uses of recorded plants were recreational tea; for jam; as snacks and soup; and drink. Interviewees also reported loss of foraging practice due to the habitat change as for example in case of caraway and chamomile. The results indicated that part of the reason for the main use of wild plants were linked to diet diversification.

**Abstract:**

Local ecological knowledge (LEK), including but not limited to the use of wild food plants, plays a large role in sustainable natural resource management schemes, primarily due to the synergy between plants and people. There are calls for the study of LEK in culturally diverse areas due to a loss of knowledge, the active practice of utilizing wild plants in various parts of the world, and a decline in biodiversity. An ethnobotanical study in a border region of Latvia, characterised by diverse natural landscapes and people with deep spiritual attachments to nature, provided an opportunity for such insight, as well as the context to analyse wild food plant usages among different sociocultural groups, allowing us to explore the differences among these groups. Semi-structured interviews were carried out as part of a wider ethnobotanical field study to obtain information about wild food plants and their uses. The list of wild food plant uses, derived from 72 interviews, revealed a high level of homogenisation (in regards to knowledge) among the study groups, and that many local uses of wild food plants are still actively practiced. People did not gather plants as a recreational activity but rather as a source of diet diversification. The results provide evidence of the importance of safeguarding ecological and cultural diversity due to high local community dependency on natural resources.

## 1. Introduction

Local ecological knowledge (LEK), including knowledge on the use of plants for food including but not limited to wild plant taxa, has historically been a key to survival for humankind [1]. LEK is a continuous, co-evolving system; it concerns the knowledge, practices, and beliefs that a local community fosters, through interactions with the environment, within a given space, and in the course of their history [2,3]. With an increase in overall wealth and centralised food security, such practices are rapidly changing (i.e., becoming more of a “trend”) [4]. Nevertheless, the United Nations Food and Agriculture Organization (FAO) [5] notes that the use of traditional practices can re-establish local food systems, while increasing socio-environmental sustainability and resilience. On a global scale, only a small fraction of edible food plants is used for human consumption, even though the number of edible plants exceeds 10,000 [5].

To improve human food security during less favourable times, it is important to understand the factors that affect the abandonment, maintenance, and valorisation (sensu [6]) of local practices. The availability of resources that form the base of LEK, as well as sociocultural factors, deserve closer attention in multinational and multilingual regions that share the same (or very similar) ecological conditions. The categorization of living things and systems is a quintessential part of environmental perception and it is fundamentally embedded into (and expressed by) language [7,8]. Recently studied factors in this area involve language [9], policy [10], and access to information [11]. Demographic aspects, such as gender [12] and age [13], also impact LEK.

The Latgale region provided us with an opportunity to gain insight on the abovementioned. The region is one of the more economically disadvantaged parts of Latvia. It is inhabited by speakers of Latgalian (a historical variant of the Latvian language [14]), and is characterized by those who practice the Catholic faith, as well as by a large proportion of the Catholic Russian-speaking population, while being a border zone to Russia and Belarus. It was also home to Old Believers (OB)—Russians and Belarusians who did not adopt the reforms of the Russian Orthodox Church in the 17th century, and were forced to emigrate to neighbouring countries [15,16]. Since the mid-17th century, OBs have inhabited the Latgale region and have maintained traditions based on a religious system of beliefs [17]. Many OBs came from the Polish–Lithuanian Commonwealth (1569–1795), the territory of present-day Belarus [18].

To date, the Latgale region has been studied from various perspectives, including: culture, e.g., [19,20,21,22]; linguistics, e.g., [23,24,25,26,27]; and ecology, e.g., [28]. Since the 20th century, in-depth research of the region was carried out by the Latgale Research Institute (in Latvian: Latgales Pētniecības Institūts). Despite this, limited data exist regarding wild plant uses within the region. Extensive work, however, was done on plant names across Latvia (e.g., [29,30,31,32]) as well as on historical traditional medicine (e.g., [33]) based on Latvian folklore. Folkloristic expeditions (e.g., [34]) and other folkloristic collections (e.g., [35]) also took place in Latgale, although without direct focus on wild food plant uses. 

The aim of the current work is to contribute to a better understanding of wild food uses—as researchers are already doing in many other parts of the world (e.g., [36,37,38,39])—among three sociocultural groups living in the same natural environment. The specific objectives of the study were to document the use of wild food plants in the Latgale region and to compare (quantitatively and qualitatively) the possible different wild food uses among three previously identified sociocultural groups living in the region, namely the Latgalians, Old Believers, and a mixed group (Russian speaking inhabitants with different belief systems). The study contributes to the discussion of diversity of plant usage in a multinational, multilingual, and multi-confessional context.

## 2. Materials and Methods

### 2.1. Research Site

#### 2.1.1. Location

**Figure 1:**
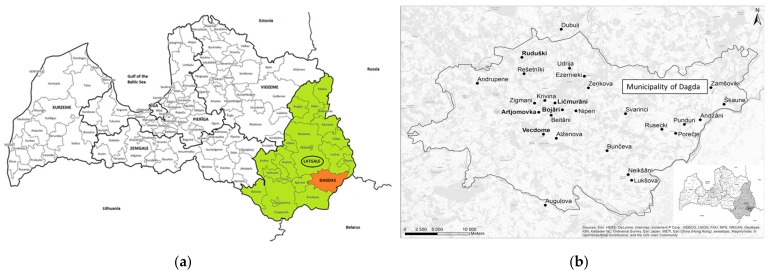
Map of the study sites (territorial division of 2016) (**a**); study sites across Dagda municipality (**b**).

The study area focused on the Dagda Municipality, which is located in the Eastern part of the Latgale region (14,000 km^2^ [40] (Figure 1). It shares a border with three countries: Russia, Belarus, and Lithuania.

Latgale is one of four regions of Latvia. Compared to the other Latvian regions, Latgale has the lowest gross domestic product per capita [41]. Latgale is also referred to as the “Land of Blue Lakes” [42]; it exhibits a diversity of landscapes that consists of fields, forestlands, and watersheds [43]. As for the Dagda Municipality, 43% of the territory is covered by agricultural land (40,226 ha), another 41% by forestland (39,190 ha), and there are 123 lakes [44]. The topography varies, from hills (e.g., in Andrupene) to flat terrains, without massive summits (maximum height not exceeding 200 metres above sea level). The soil in the area of interest is characterised as Podzolic. The Dagda Municipality has a continental climate, with an average yearly temperature of 5.1–5.2 °C and an average rainfall of 650–700 mm. The vegetation period lasts 136–140 days [44]. 

We selected this study site because of its diversity of cultures, including the restrictions of interactions between different sociocultural groups over centuries. A clear example of the diversity of the region is its ethnic composition. The Dagda district consists of Latvians (62%), Russians (25%), Belarusians (7%), Poles (3%), and representatives of other nationalities (3%) [45]. Part of the population has a hybrid identity [46], particularly individuals born into mixed families and speaking mixed languages. Still, the secluded lifestyle of the OB community [47] reduced the contact between cultures. However, interaction was possible in villages between Latgalians and OBs (e.g., according to the written sources, such as inventories); 10 of 119 villages documented in 1765 were inhabited by OBs and Latgalians [18].

Historical events altered the borders of the study site several times. For example, during the 19th century, the current Dagda Municipality belonged to the Vitebsk Governorate of the Russian Empire (Витебская губерния), which covered nearly the entire Latgale region [48]. Then, beginning in 1920, Dagda belonged to Daugavpils and Rēzekne Municipality (apriņķis). After a change in administrative territories in 1947, part of the present-day Dagda Municipality was divided between two municipalities: Krāslava and Rēzekne, where all parishes, apart from Dubuļi, were part of the former. From 1950 to 2009, it was called Krāslava District [49].

The interviews took place in 27 villages located within a distance of 28 km. While the Old Believers were all quite closely situated (Artjomovka, Bojāri, Ličmurāni, Vecdome, and Ruduški, which even has its own congregation of OBs), the rest of the interviewees were spread out within the historical administrative division, apart from Dubuļi Parish.

#### 2.1.2. People, Language and Culture

From 2010 to 2018, the number of inhabitants in Dagda Municipality shrank from 9331 to 7361, of which 4673 were Latvian citizens, including 3644 men and 3717 women [50,51]. As the Latgale region is (economically) poorly developed, with a high unemployment rate and unreliable infrastructure, the population has shrunk in the last few decades [52].

The basis of a unified Latvian language had formed by the 16th century. It belongs to the Baltic group of the Indo–European family of languages [53]. According to Latvian Language Law, Latgalian is a historical variety of the Latvian language [14]—the High Latvian dialect (here after Latgalian dialect) that developed separately over several hundred years and was influenced by the Polish language, especially in its written form, during the rule of the Polish–Lithuanian Commonwealth, until the 1830s [54]. Since the 18th century, Latgalian was influenced by the Russian language spoken by inhabitants belonging to the Orthodox Church and OB community [27].

The most intensive Russification took place from 1865 to 1904, when the Latgalian language was banned in its written form [54]. Up until the 20th century, Latgalians lived in villages, resisting Russification [55] and transferring knowledge from generation to generation [18]. However, the influence of the Russian language was observed by folklore collector Pēteris Gekišs from Dagda in 1927: “the Latgalian language is heavily mixed with foreign words, especially with Russian in Dagda Parish. A purer language is used by elderly people only” [35]. 

After the establishment of the Republic of Latvia in 1918, Latvian became the official language. Latgalian was used as the official language in the Latgale region; however, its usage has been limited since 1934. During the Soviet Union (1940–1991), Latgalian survived in its spoken form (mainly in church), although this is not standardised. According to data from 2011, approximately 8% of Latvian residents (164,000 individuals) speak Latgalian on an everyday basis [56]. Of those residents who use Latgalian regularly, three-quarters primarily use Latvian at home and one-quarter uses Russian [57].

Latgalian is used on an everyday basis in the Latgale region (one-third of the population uses Latgalian) by 71% of the Latvian-speaking and 12% of the Russian-speaking inhabitants [56]. However, Latgale may be considered trilingual as people speak Latvian, Latgalian, and Russian [58]. Latvian citizens of Slavic origin often are Russian speakers, though they are not Russian [59]. This reflects the multicultural environment of this region [60], in terms of ethnicity, language, and religious and cultural traditions [21,61,62,63]. As analysed by Ivanovs and Soms [64], the characteristics of the Latgale region have been framed by Latgalian mentality through language and influence from neighbouring countries, in addition to other factors, such as customs and lifestyles, and the languages of other nations. 

### 2.2. Data Collection

The data were collected by conducting semi-structured interviews, which lasted around 0.5–2.5 h per person, in July 2017. The fieldwork was part of a larger study investigating the ethnobotanical aspects of the region, with a focus on wild plant uses as food. The interviewees were first asked to free-list wild plant species used as food and then to name the wild plants they (or their family members) use now, or have used in the past, for preparing specific food categories (e.g., soup, pie, jam, desserts, compote, spice, salad, snacks, tea, and beverages). Past uses referred to wild plant uses no longer practiced, e.g., uses from childhood, while those still practiced today were defined as current uses.

The interviews were conducted following the guidelines of ethnobotanical research and approaches used in similar studies (e.g., [65,66,67,68,69]). As part of the ethnobotanical fieldwork, a walk in the vicinity of the garden was conducted, whenever possible, with the interviewees, and they were asked to point to various species they know and use.

Interviews were conducted in either Latvian or Russian, depending on which language the person was most comfortable with (the interviewing team was bilingual). Several of the people interviewed in Latvian were multilingual, but Latvian was their preferred language.

Social research methods were used when select participants (via convenience and snowball sampling approaches) [68,70]. Snowball sampling is particularly useful when conducting research in scattered populations (see [71]). The information was gathered from 73 local interviewees, of which 72 reported plant food uses, including 26 males and 46 females. The small sample of male interviewees was due to women being more readily available for conversation, especially among the Russian-speaking community. The average age of the interviewees was 63 years, with a maximum of 81 years and a minimum of 30 years.

The mechanisms, as well as the pattern of plant use practice, affected the nomenclature of the taxa used. Therefore, we devoted attention to single (one time mention) uses as well. First, a single use may reveal traces of historical use. Second, it can provide important insight into how a practice was formed. While historically every person had their own collection of plants for certain application, the communal herbal landscape (sensu [72]) was quite “even”, and, more or less shared, and the selection of used plants was limited among laypeople (see, for example, [73]). Single uses in modern data, especially if they are numerous, can show patterns in regards to the adoption of knowledge and the individualization of a practice once shared within a community [69]. 

The study followed the Code of Ethics of the International Society of Ethnobiology [74]; verbal, informed consent was obtained from all interviewees. The interviews were recorded upon permission of the interviewees for the purpose of transcription only.

### 2.3. Plant Specimen Collection and Identification

As part of the fieldwork, during the interviews, plant specimens were collected, pressed, dried, and identified by the third (I.M.) and fourth (R.K.) authors (following [67,69]). Plant taxonomic identification was based on local flora [75]. Identifications were checked by Toomas Kukk, curator of the Estonian University of Life Sciences herbarium. Specimens were deposited at the Estonian University of Life Sciences herbarium (TAA), bearing numbers LGA001-120 and herbarium numbers TAA0146373–495. The authors collected dried plant samples offered by the interviewees (deposited at the Herbarium of DAIS at Ca’ Foscari University of Venice (UVV), bearing numbers UVVDLGA001–71). Specimens are available for public study at both institutions. The authors followed The Plant List database [76] for plant taxa names; the Angiosperm Phylogeny Group IV [77] was used as a reference for plant family names. The names of the collective plant species follow Flora Europaea [78].

### 2.4. Data Analysis

All interviews were transcribed from recordings and notebooks and the data were entered into a Microsoft Excel spreadsheet, according to interviewee-defined food categories. Use Instances (UI—the detailed use-report regardless of the number of people mentioning a particular use), calculated for comparisons, were derived from emic food categories.

Forty-one wild food use-categories were identified. The data was further structured in detailed use-reports (DUR), reflecting the use of a plant part prepared or applied in a certain way for a certain food category, multiplied by the number of people mentioning such a use. In exceptional cases, cultivated plants were also included in the discussion if they had not been cultivated for food purposes, or if the part used was not regularly used as food (e.g., the leaves of *Prunus avium* L., *Borago officinalis* L. and *Bergenia crassifolia* (L.) Fritsch).

### 2.5. Comparisons

Components forming the sociocultural identity of Latgale inhabitants are determined by historical and political events, religion, and the interaction among people of different ethnicities and languages [21,79]. Therefore, for comparative purposes, people were assigned to one of three groups (Table 1) based on the following criteria:

(a) OB group: study participants who identified themselves as Old Believers, spoke Russian, and followed the faith of Old Believers’ Eastern Orthodox Christianity;
(b) Latgalian (LG) group: interviewees who were Catholic, spoke fluent Latvian and/or Latgalian, chose Latvian as the preferred language of communication, and claimed to come from the Latgale region, even if, on the rare occasion, one of their parents was of non-Latgalian origin;
(c) Mixed (MIX) group: the remaining interviewees were assigned to the MIX group, which consisted of people who had very diverse ethnic, linguistic, and spatial origins, but chose Russian as the preferred language for the interview (e.g., people of Polish, Russian, and Belarussian origin, with different belief systems, apart from that of Old Believers). Considering the complexity of this particular group, in terms of the variables mentioned above, as well as the limited number of interviews, it was not possibility to divide the group into smaller units. The group represents the multinational and multilingual constituency of the population of the region.

**Table 1:** Information on the interviewees.

Language Group	LG		MIX		OB	
Gender	f	m	f	m	f	m
Number of people	16	11	21	5	9	10
Total number (^^^ only mentioned past uses) of interviewees naming food uses	27 (^^^ 1)		26 (^^^ 1)		19	
Average age	62	66	66	63	63	54
Main religion	Catholic		Catholic/ non-believer	Catholic/ Orthodox	Old Believer	
Educational composition	pf				pf/he	pf
Origin	Autochthonous		Local/from the neighbouring area (~56%); various, including arrival during the Soviet era, e.g., 1959		Mass settlement in the Baltic region during the second half of the 17th century *	
Languages spoken by parents	Latgalian/Latvian (80%), Russian/Latvian/Latgalian (12%), Belarusian (8%)		Russian/Latvian/Latgalian (41%), Belarusian/Russian/Latvian (27%), Latgalian/Latvian (9%), Polish/Latvian (23%)		Russian	

Abbreviations: LG—Latgalian; MIX—Russian-speaking population with mixed background; OB—Old Believers; pf—professional education; he—higher education; * [80].

Some species that botanically belonged to the same genus, but were treated as a single taxon by interviewees (the same name and same uses, and/or did not distinguish species in the field), were gathered into a species pluralis (also for related calculations). Some genera, e.g., *Hypericum*, *Trifolium*, *Urtica*, *Betula*, *Rumex*, *Matricaria* and *Mentha*, were identified on the genus level due to the overlap of the local names used in the region. While comparing the three sociocultural groups, we excluded all uses described as practiced in the past from the comparative analysis. 

In addition, the authors compared UIs and taxa recorded for all three groups. Jaccard Similarity Indices (JI) were calculated for used taxa following González-Tejero et al. [81]: JI = (C/(A + B − C)) × 100 where A represents the number of taxa in sample A, B is the number of taxa in sample B, and C is the number of taxa common to A and B. 

## 3. Results

In total, 75 plant taxa belonging to 38 families were recorded as used as food. The most commonly used were Rosaceae (12 taxa with 193 DUR), Ericaceae (4/184), Polygonaceae (1/73), Betulaceae (4/72), and Apiaceae (3/53). Of the taxa, the most commonly used was *Vaccinium myrtillus* L. (83 DUR), followed by *Fragaria vesca* L. (74), *Rumex* spp. (73), *Rubus idaeus* L. (69), and *Carum carvi* L. (49). 

The most represented current uses were recreational tea (137 DUR), jam (122), snacks (108), soup (71), drinks (63), condiments for pickles (57), as salad (36), for smoking meat (30), and desserts (26). Table 2 shows an overview of the food uses of plants in the Latgale region (both past and present applications).

### 3.1. Past and/or Peculiar Uses

Among all used taxa, a total of 45 species were mentioned as being used in the past, of which 12 were used solely in the past, as noted by several people. From the rest, 31 taxa were used in the past, of which three (*Carum carvi*, *Fragaria vesca*, *Vaccinium oxycoccos*) had 10 or more past use records. The main past uses named by interviewees were snacks (24 DUR), recreational tea (20), and jam (12). The proportion of taxa only used in the past among all uses was just 16%. Most of the past uses no longer practiced were recoded among the Latgalian group and mentioned by only one or two people. For example, one woman (b. 1951) recalled eating the tubers of *Equisetum arvense* in her childhood, stating, “They tasted like nuts”. Her husband added, “You were like a cow, eating everything”. She also remembered a tea made by her grandmother—the leaves of *Malus domestica* were placed in a clay pot and steamed in a hot oven, then dried and used as a delicious recreational tea.

Only two interviewees referred solely to past uses. One of them, a man (b. 1942), described his wife as the main collector of plants; after her death, he did not continue the practice. Other past food uses were also been historically used in neighbouring regions. A Russian-speaking man (b. 1940s) of Belarusian origin recalled the use of shoots of *Heracleum sphondylium* as food in the hard times, a widely known practice in the 19th century [82]. 

Some past plant uses, mostly snacks, were related to childhood. An interviewee (b. 1948) of Polish origin recalled that children were forbidden to eat nuts (*Corylus avellana*) until Christmas: “Nuts, carrots and apples—there’re children’s food.” A similar belief was also recorded in the territory of Poland [83]. Moreover, the use of nuts as Christmas candy was recorded in Estonia [84]. 

There were also a few reports on the fermentation of *Beta vulgaris subsp. vulgaris var. vulgaris*. One of them was from the distant past, 50 years ago (beetroots were fermented with rye flour, then ground and added to soup), while another was a recent trial, made just once (both reports by OBs). In addition, one OB and one Russian-speaking Catholic reported making kvass in their childhood. The OB also reported a recent trial of making kvass from *Beta vulgaris subsp. vulgaris var. vulgaris*, but he claimed it was not successful. Only one Latgalian, a woman (b. 1956), recalled the memory of her father using *Beta vulgaris subsp. vulgaris var. altissima Döll* for making *moonshine*.

### 3.2. Comparison of Current Uses among the Groups

The current use of 63 plant taxa belonging to 34 families was recorded. The most commonly used were Rosaceae (11 taxa with 171 DUR), Ericaceae (4/164), Polygonaceae (1/69), Betulaceae (4/58), Asteraceae (8/39), and Apiaceae (2/36). The taxa currently used the most for food were *Vaccinium myrtillus* (76 DUR), *Rumex* spp. (69), *Fragaria vesca* (64), *Rubus idaeus* (63), *Vaccinium vitis-idaea* (44), and *Betula* spp. (41).

**Figure 2:**
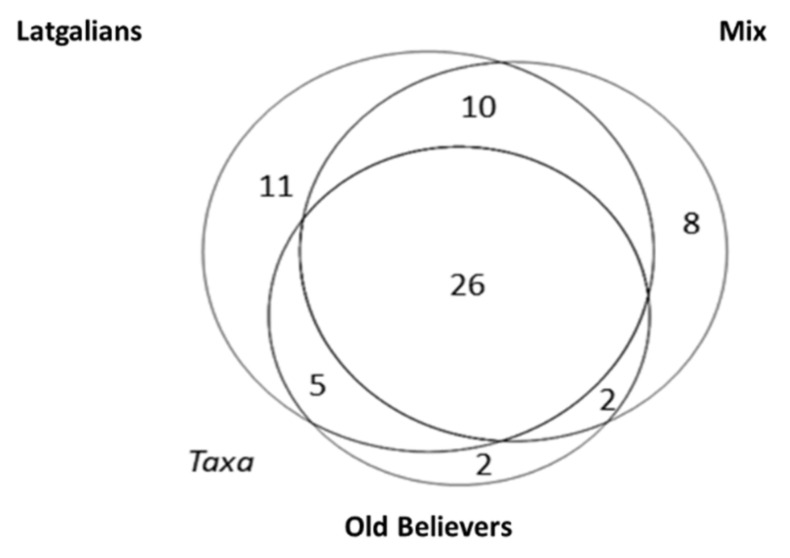
Overlap of taxa among the three sociocultural groups: JI Latgalians—Mix: 58%, JI Latgalians—Old Believers—55%, JI Mix—Old Believers—53%.

The most diverse sociocultural group, in terms of taxa used, and uses, was that of Latgalians (Figure 2, Table 3). OBs stand out by exhibiting the lowest number of taxa used, while sharing relatively similar taxa and uses with the other two groups.

**Figure 3:**
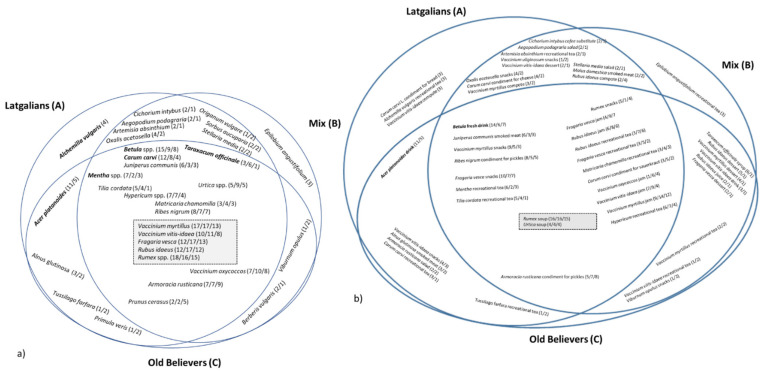
Taxa (**a**) and UIs (**b**) currently most used by the three different groups.

The number of interviewees per used taxon shows a similar tendency, but even less difference between the groups (Figure 3).

In the overlapping area of all three groups, we included taxa only used by at least 10 people total, for which the number of people reporting uses are presented as (A/B/C). For the taxa used solely by one or two groups, only taxa with at least three users are presented, for which the number of people reporting uses are provided as (A/B), (A/C), and (B/C), respectively. The grey area in the centre highlights the most widely used taxa/UIs, which are most equally utilised. The names in bold represent borderline taxa used predominantly in one region.

The only taxon used exclusively by one group by more than three people was *Alchemilla vulgaris*, which was used by Latgalians for recreational tea. The other differences are represented by three or fewer users, with a few exceptions, where the plant, or specific uses, were more often mentioned within one of the groups. The tendency to name wild and traditionally used plants seems characteristic of Latgalians, while OBs were keener to mention plants related to cultivation (such as *Armoracia rusticana*); in the mixed group, the plants were promoted by the literature (such as *Taraxacum officinale* and *Epilobium angustifolium*). For Old Believers, it is important that food and drink be kept apart from the external “pagan” world to maintain the natural purity [85].

### 3.3. Latgalian Plant Names—A Marker of Cultural Diversity

Interviewees mentioned 13 local plant names in Latgalian. Most of them were used for recreational tea (eight: *pelešķi, duobuls, ūzūls, līpas, prīdes, goilīņes, gaiļpieši, topoļi*) and snacks (five: *aveņis, skuobines, zaķskuobines, duobuls, airi*). Of all Latgalian plant names, one is borrowed from Russian—*topoļ* (*Populus* spp., *тoпoль*) as the plant was introduced into Latvia and had no vernacular name. *Aveņis* (*Rubus idaeus)* is a Latvian name used with Latgalian pronunciation and would be called *avīkšas* in Latgalian. The importance of referring to plants “correctly” (in Latvian instead of Latgalian) was stressed by some of the interviewees (e.g., woman, b. 1965), reflecting the attitude that vernacular names were less important in communication even if they were used within the family.

The manner in which the use of food plants has been affected by other cultures is reflected in lexical items—interviewees speaking Latvian and Latgalian used plant names borrowed from Russian, such as ļebeda, sņitka, kmins, hrens, zveraboj, romaška, golubika, brusiņika, etc.

## 4. Discussion

The greatest number of wild food taxa in the region was recorded in Saaremaa, Estonia (89, [86]), located north of Latgale, followed by more southerly regions, including Herzegovina (82 taxa; [87]) and costal Croatia (80 in Poljica and 76 in Krk; [88]). The number of recorded taxa (75) in this study is slightly lower. Still, even the number of taxa used by one of the groups is higher than the numbers recorded in areas to the south of the region, where the number of taxa available for food could be higher: 26 taxa used in Roztochya [89], 35 taxa used by Boikos [90], 40 taxa used by Hutsuls [10], and 44 taxa used by Ukrainians in Maramures [91]. The total number of uses is also higher compared to the 58 taxa used in central Belarus [69], and that mentioned by Rostafinski’s interviewees from Belarus in the 19th century [92], as well as the 55 taxa used on the Dubrovnik coast [93]. The similarities among the taxa used by different groups in our study are fewer than in the case of the Boikos and Hutsuls (69% overlap for taxa; [90]), yet the overlap of uses is among the highest ever recorded among different linguistic and/or cultural groups. The most commonly used families in this study follow the pattern observed in other studies conducted in Eastern Europe. 

Filosofova [80] considered the Latgalian OB community as an example of preservation of one’s own original culture in a foreign environment. However, this is not reflected in the wild food domain, as the OB group did not have any specific wild food uses unique to them and used by more than three people. The OBs also relied on cultivation, e.g., *Beta vulgaris*, *Prunus cerasus* (see [85]).

By analysing lexical items of OB communities in Siberia, it is obvious that the names of bread and cereal dishes are used more often (38%) [94]. This reflects the significance of cultivated and nutritious food sources in OB culture. Even some cultivated plants, such as potatoes, horseradish, onions, and garlic, were originally prohibited in some Eastern Slavic communities [95], as were imported, foreign plants, which were considered sinful and cursed by Satan, but were used to make intoxicating or stimulating drinks (*Humulus lupulus*, coffee, black tea) [96]. They were replaced by local wild plants [97].

It was observed by several authors that, during Soviet times, OB culture became more open to external influences (mostly because of centralisation policies that contributed to cultural admixture and Russification) [47,55,98]. Over time, the plants were adopted into OB culture; for example, in neighbouring Estonia, onions and cucumbers were grown and sold in Leningrad (St. Petersburg) during the Soviet era [99]. However, the number of wild plants used as recreational tea among Russian OBs of Estonia was limited: nowadays they are used as an additive to oriental black tea [100].

As some interviewees admitted, creating salads from wild greens is most likely of very recent origin, learned from books, newspapers and, now, social media. OBs, however, did not have such uses. Yet, for some interviewees, the plants were noted as a source of famine food during hard times, e.g., *Heracleum sphondylium subsp. sibiricum, Rumex* spp. and *Oxalis acetosella*. An interviewee (Belarusian woman b. 1940) expressed strong emotions and became bitter while remembering that she was forced to collect wild greens in order to survive during and after World War II. 

Some of the interviewees expressed a preference for a particular taste. For example, a Latgalian woman (b. 1959) noted that she collects wild species of *Rumex* spp. due to their taste (as garden sorrel is too sour). Changes in taste were also perceived by a Polish interviewee (woman, b. 1949) who mentioned that none of her family members liked jam anymore, and for that reason, she only uses frozen berries.

Homogenisation of society during the times of the Soviet Union contributed to unification of LEK (see [101,102,103]), where the educational system, which is characterised as particularly centralised [104], serves as one contributing factor. As ascertained by Strods [105], Russification of schools and public life of the Latgale region increased the number of Russianized families. Several interviewees noted that they studied in schools, in Russian, as there were no schools in Latvian available in their village, and they now prefer to speak Russian instead of Latvian (see [106], etc.). A Polish interviewee (b. 1948), who speaks a mixture of Polish and Belarusian in her family (as her husband is from Belarus) and Russian outside the home, claimed that she could not clearly indicate her own ethnic identity. In contrast, some of the interviewees stressed that they are able to speak several languages, but related their ethnic identities to their mother tongues (language spoken in their family during childhood). For example, a man (b. 1942) stated that he can speak Latvian, Latgalian, Russian, and Belarusian, but he considered himself Latgalian. Likewise, a woman (b. 1957) who spoke Russian in her family, and during the interview, considered herself Latgalian because of her native language.

However, integration of the use of *Epilobium angustifolium* as a recreational tea by the mixed group alone is interesting; such a use is a trend that has spread in the region recently (although one OB recalled it as a past use (see also [107]). However, a study reflecting on the historiography of Latgale by Ivanov [21] notes the term “tradition of co-existence” (p. 9). In various ways, this is also reflected in wild food plant uses, with high overlap between different sociocultural groups. 

Change in plant uses has a certain connection with landscape change. For example, *Carum carvi* and *Matricaria chamomilla* are now often bought from the shop, as their places of growth have disappeared, or people can no longer find them in the wild. *Matricaria chamomilla* is now only found in the wild when it escapes from the gardens and it cannot survive in nature for more than a few years. Culturally, *Vaccinium oxycoccos* is the most important berry, which grows further from human pathways, but at the same time, only two respondents mentioned *Rubus chamaemorus*. Here, the importance of local knowledge enables local community members to support their livelihoods. Remarkably, the majority of the interviewees from the Dagda region did not gather plants as a recreational activity, but rather as an additional food source, as is the case in other peripheral areas in Europe (most notably in the Balkans [108,109,110]) and Estonia [102]. In our field study, we did not find any evidence of a recreational aspect of foraging as found in Southwestern and Central Europe [4,111,112]. As pointed out by scholars [89], the consumption of wild food, particularly non-wood forest products, is linked to the socioeconomic aspects in the country of interest. For example, in Finland, wild berry picking is considered part of a rural lifestyle [113]. Moreover, local gastronomic diversity is of great importance when talking about food security (see [114]). 

## 5. Conclusions

The results of this study, conducted in the highly multinational (numerous ethnic groups), multilingual, and multi-confessional (different religions) region of Dagda Municipality, illustrate the significant overlap in LEK within three groups, differing either linguistically or religiously. In our sample, the LG group used more species in total; however, this may be due to the uneven sample sizes (compared to, for example, the OB group). However, all study groups have kept practices alive as considerable proportions of all recorded uses are still actively practiced. The mixed group of people who preferred to speak the *lingua franca* of Soviet times was keener to accept teachings from the media and literature. In addition, the biodiversity (and provisional ecosystem services in general) of nearby grasslands and forests have high local value in supporting the community with the basic needs of additional nutrition sources. The active use of local resources targeted in situ and ex situ conservation actions, as already proposed by other authors in many European and Mediterranean natural ecosystems and agroecosystems (e.g., [115,116,117,118]). This serves as proof that a wide base of LEK in the community exists and the narratives expressing the loss of certain taxa serve as arguments supporting the need for local resources. This on the other hand gives a ground for the need to begin a rapid action on sustaining the plant food uses.

This study also provides possible input for formulating a hypothesis in regards to the effects of religion on LEK. Future research should also investigate the possibilities of developing small-scale enterprises that specialize in the production of wild food products with added value; thus, increasing the employment rate and social security in a region. Furthermore, the authors foresee a need to integrate various strategies, e.g., foraging as part of school curriculum in order to celebrate biocultural diversity. 

## Figures and Tables

**Table 2:** Food uses of plants in Dagda Municipality.

Scientific Name	Family	Herbarium Number	Local Names	P, C or W	Used Part(s)	Preparation	Uses	LG	MX	OB
*Acorus calamus* L.	Acoraceae		airi *	W	spadix	fresh	snacks	1		
					leaves	fresh	under bread	/1		
					rhizomes	fresh	snacks	/1		
*Viburnum opulus* L.	Adoxaceae		калина	W	fruits	cooked	jam			1
						fresh	snacks		1	2
*Atriplex* sp.	Amaranthaceae		лебеда	W	aerial parts	fresh	condiments for salad		/1	
							soup			/1
^@^*Beta vulgaris* subsp. *vulgaris* var. *vulgaris* L.	Amaranthaceae		бурак, бураки красные, biete	C	roots	fermented	eaten			/1
							moonshine	/1		
							kvass		/1	/1
						fermented with rye flour	ground, added to borsht			/1
*Chenopodium album* L.	Amaranthaceae	LGA008, LGA037	balanda, ļebeda ^	W	aerial parts	fresh	salad	2		
*Allium schoenoprasum* L.	Amaryllidaceae		maurloki	W ^©^	leaves	fresh	salad	1		
*Allium ursinum* L.	Amaryllidaceae		чeрeмша, lakši	W ^©^	leaves	fresh	salad	1	1	
*Aegopodium podagraria* L.	Apiaceae		sņitkas ^, gārsa, шнитка, снить, gārses	W	leaves	fresh	salad	2	1	
*Carum carvi* L.	Apiaceae	LGA061	тмин, ķimene, kmins ^, tmin ^, ķimene, savvaļas ķimene, кмин	W	seeds	dried, fresh	condiment	1	2	
							condiment for bread	3/3		/2
							condiment for cheese	5/1	2	
							condiment for sauerkraut	3/1	5/2	2/2
							condiment for meat	1	/1	
							condiment for sausages	1	/2	
							condiment for soup	2	1/1	
							recreational tea	3		1/1
							added to vodka			1
*Heracleum sphondylium subsp. sibiricum* (L.) Simonk.	Apiaceae		гигельник	W	shoots	not remembered	food in hard times		/1	
*Achillea millefolium* L.	Asteraceae	DLGA054	pelašķis, pelašķi, тысячелистник	W	aerial parts	dried	recreational tea	2	3	2
*Artemisia absinthium* L.	Asteraceae		vērmeles, poliņ ^, пoлынь	W	aerial parts	dried	recreational tea	2	1	
*Cichorium intybus* L.	Asteraceae		cigoriņš, цикoрий, cigoriņi	W	roots	dried	recreational tea	1		
						roasted	coffee substitute	2	1	
*Helianthus tuberosus* L.	Asteraceae		topinambūrs	C	tubers	fresh	condiments for salad	1		
*Matricaria* spp. including *Matricaria chamomilla* L., *Matricaria discoidea* DC	Asteraceae	DLGA048	kumelīte, romaška ^, рoмашка	W	flowers	dried	recreational tea	4/1	4	3/1
*Taraxacum officinale* (L.) Weber ex F.H. Wigg.			oдуванчик, pienenes	W	flowers	cooked	syrup		7	1/2
						fresh	snacks		1	
					leaves	fresh	salad	2		
					aerial parts	cooked	condiment for soup		/1	/1
*Tussilago farfara* L.			мать-и-мачеха, māllēpe, mač i mačiha ^	W	flowers	dried	recreational tea			1
					leaves	cooked	soup		/1	/1
						dried	recreational tea	1		1
*Berberis vulgaris* L.	Berberidaceae		барбариса, барбарис	W	fruits	cooked	jam		1	1
							juice		1	
*Alnus glutinosa* (L.) Gaertn.	Betulaceae		melnais alksnis, melnais elksnis, oļha čornaja ^, oльха черная, черная oльха	W	wood	burned	smoking meat	4/1		2
*Alnus incana* (L.) Moench			oльха, alksnis	W	wood	burned	smoking meat	2/2	2/1	2
*Betula* spp. including *Betula pendula* Roth, *Betula pubescens* Ehrh. and their hybrids			берёза, bērzs	W	leaves	dried	recreational tea	1		
						fresh	salad		1	
					sap	fermented	kvass	2/1	4	2
							wine	/1		
						fresh	drink	14	6/1	7/1
						frozen	drink	1		
						stored with citric acid and sugar	drink	1		
					wood	burned	bread baking			1
							smoking meat	1		
*Corylus avellana* L.		LGA005A	lazdas, oрехи лесные, oрехи, oрешник	W	nuts	fresh, dried	snacks	2/4	2/1	1/1
*Borago officinalis* L.	Boraginaceae		gurķumētra, gurķa mētra	C	leaves, aerial parts	fresh	salad	2		
						fresh, frozen	soup	2		
*Armoracia rusticana* P. Gaertn., B. Mey. and Scherb.	Brassicaceae	LGA056	mārrutks, хрен, mārrutki, hrens ^	C/W	leaves	fresh	condiment for pickles	2/1	1	4
							salad	1		
					roots	fresh	condiment for meat		1	
							condiment for pickles	3	8/1	8
							salad	1		2
*Humulus lupulus* L.	Cannabaceae	LGA029	apinis	W	cones	dried	taste additive to beer	/2		
*Valeriana officinalis* L.	Caprifoliaceae		валерианка	W	roots	dried	recreational tea		2	
*Stellaria media* (L.) Vill.	Caryophyllaceae	LGA009, LGA040, LGA070	virza, мoкрица, снитка	W	aerial parts	fresh	salad	2	2	
*Juniperus communis* L.	Cupressaceae		верес, мoжжевельник, paeglis, kadiķis, верeсoк	W	twigs	burned	smoking meat	6/2	3	3
*Equisetum arvense* L.	Equisetaceae		tīruma kosa	W	tubers	fresh	snacks	/1		
*Vaccinium myrtillus* L.	Ericaceae	LGA007, DLGA043, DLGA061	черника, mellenes, чoрные	W	fruits	cooked	compote	3	2	/1
							jam	9/1	14/1	12/2
							drink	1	3	1/1
						cooked, frozen	dessert		6	1
						dried	recreational tea		1	1
						fresh, frozen, dried	snacks	9	6/1	4
					aerial parts	dried	recreational tea		1	2
*Vaccinium oxycoccos* L.			клюква, dzērvene, dzērvenes	W	fruits	cooked, frozen	jam	1/1	6	4/1
						fresh	compote			2
							condiment for sauerkraut	1/1	1/1	/1
						fresh, frozen	dessert	2	3/1	1
							drink	1	5/2	2
							snacks	2/2	1	1
*Vaccinium uliginosum* L.			zilenes, гoлубика, golubnika ^, golubika ^, пьяница	W	fruits	cooked	compote	1	1	
							jam	3	1	1
						fresh	snacks	1	2	
						juice	juice		1	
*Vaccinium vitis-idaea* L.		LGA004, DLGA069	брусника, brusiņika ^, brusnika ^, brūklenes, журавина, brūklenājs, брусничник	W	fruits	cooked	compote	3		
							drink		3	1
							jam	7	9/2	4
							jam with apples	1	1	1/1
							pie with berries		1	
						fresh, frozen	snacks	3		3
						frozen	dessert	2	1	
					leaves	dried	recreational tea		1	2
						fresh	snacks	1		
*Quercus robur* L.	Fagaceae		ozols *, ūzūlu *, дуб	W	acorns	dried	condiment for malt	/1		
							recreational tea	/1		
						fresh	added to vodka	/1		
						roasted	coffee substitute	/1		
					buds	tincture	drink		/1	
					leaves	fresh	condiment for pickles	1		1
*Ribes nigrum* L.	Grossulariaceae	LGA112	upene, черная смoрoдина, upenes, melnās upenes, смoрoдина, черная смoрoда, смoрoдина черная	C	twigs	fresh	condiment for birch sap	2/1	1	1
							condiment for pickles	8	5/1	6/1
							preservative for sap		1	
							recreational tea			2
*Ribes rubrum* L.	Grossulariaceae		красная смoрoдина	C	leaves	dried	recreational tea			1
					twigs	fresh	condiment for pickles			1
*Philadelphus coronarius* L.	Hydrangeaceae		жасмин, jasmīni	C	flowers	dried	recreational tea	1	1	
*Hypericum* spp. including *Hypericum maculatum* Crantz and *Hypericum perforatum* L.	Hypericaceae	LGA027 DLGA030	зверoбoй, зверoбoи, zveraboj ^, asinszāle	W	aerial parts	dried	recreational tea	6	7/2	4/1
							added to vodka	/1		
						fresh	added to vodka	1		
*Mentha* sp., including *Mentha longifolia* and *Mentha suaveolens*	Lamiaceae	DLGA035	мята, istabas piparmētra, mjata ^, piparmētra, polan mjata ^, savvaļas piparmētra, piparmētra, mētra	C	leaves	fresh	snacks	/1		
					aerial parts	cooked	syrup	1		
						dried	condiment for soup	1		
						fresh, dried	recreational tea	6	2	3
*Origanum vulgare* L.		LGA031	душевица, душица, raudene	W	leaves	fresh	salad	1		
					aerial parts	dried	recreational tea		2/1	
*Salvia officinalis* L.			матушник	C	leaves	dried	condiment for bread		/1	
*Thymus serpyllum* L.			mārsils, чaбрец	W	aerial parts	dried	recreational tea		1/1	
						fresh	condiment for pickles	/1		
*Trifolium* spp. including *Trifolium medium* L., *Trifolium pratense* L.	Leguminosae	DLGA013, DLGA038, LGA034, DLGA026	sarkanais āboliņš, āboliņš sarkanais, клевер, dāboliņš, клевер дикий красный, клевер красный, sarkanais duobols *, sorkonais doubuls *, duobuls *	W	inflorescences	dried	added to bread		/1	
						fresh and dried	recreational tea	1/1	1	/1
							snacks	1		1
*Trifolium repens* L.			baltais āboliņš	W	aerial parts	dried	recreational tea	2		
*Tilia cordata* Mill.	Malvaceae	DLGA009, LGA011	liepa, липа, līpas *	W	inflorescences	dried	recreational tea	5	4/3	1
*Epilobium angustifolium* L.	Onagraceae		иван-чай	W	aerial parts	dried	recreational tea		3	/1
*Melampyrum nemorosum* L.	Orobanchaceae		puķītes dzeltenas ar zilu (nārbulis)	W	flowers	fresh	snacks	/1		
*Oxalis acetosella* L.	Oxalidaceae	LGA122	заячья капуста, zaķskābenes, zaķa skābenes, zaķskuobines *, zaķkāposti, заячья кислица	W	aerial parts	fresh	food in hard times		/1	
							snacks	5	2	/2
*Oxalis corniculata* L.		LGA063	zaķskābene	W	leaves	fresh	snacks	/1		
*Pinus sylvestris* L.	Pinaceae		coснa, prīdes *	W	buds	dried	recreational tea	/1	1	
*Plantago major* L.	Plantaginaceae		пoдoрoжник	W	leaves	cooked	eaten		1	
*Phleum* sp.	Poaceae		тимoфеевка	W	stems	fresh	snacks		1	
*-*	*Poaceae*		parastā smilga	W	stems	fresh	snacks	1		
*Rumex* spp. including *Rumex thyrsiflorus* Fingerh., *Rumex acetosa* L.	Polygonaceae	LGA086, LGA064, LGA036, LGA121	parastā skābene, skābenes, skābene, щавель, кислица, pļavas skābenes, savvaļas skābene, skuobines *, zirgu skābenes	W	leaves	cooked	soup	19/1	17/2	20
						fresh	salad	2	/1	
							snacks	5	1	4
						frozen	soup	1		
*Primula elatior* (L.) Hill	Primulaceae	LGA091, LGA121, DLGA027	gaiļpieši ^^, goilīņes *, петушки	W	inflorescences	dried	recreational tea	1		
					leaves	fresh	salad	1	1	
*Primula veris* L.			первoцвет, gaiļpieši	W	inflorescences	dried	recreational tea			2
					leaves	fresh	salad	1		
*Frangula alnus* Mill.	Rhamnaceae		krūklis	W	fruits	fresh	snacks	1		
*Alchemilla vulgaris* auct. (coll.)	Rosaceae		raspodiņš, rasas krēsliņš, rasenes, rasaspodi, raspodiņi	W	inflorescences	dried	recreational tea	2		
					leaves	fresh	salad	1		
					aerial parts	dried	recreational tea	1		
*Crataegus* sp.			бoярышник	W	fruits	cooked	jam		1	
						fresh	snacks		1	
*Fragaria vesca* L.			meža zemene, земляника, meža zemenes, zemļenica ^, zemļaņika ^	W	fruits	cooked	jam	5/2	9	7
							juice	1		1/1
						fresh	compote	/1	1	/1
							dessert		2	
							snacks	11/2	6/1	7/1
						frozen	dessert			1
							snacks	2	1	
					leaves	dried	recreational tea	1	1	
					aerial parts	dried	recreational tea	2	4	1
						fresh	recreational tea			1
*Malus domestica* Borkh.			ābele, jablonja ^, яблoня	C	leaves	dried	recreational tea	/1	2	
					wood	burned	smoking meat	2	2	/1
							smoking meat and fish			1
*Malus* sp.			яблoки лесные	W	fruits	dried	recreational tea			1
*Prunus avium* L.			višņi, ķirši	C	leaves	fresh	condiment for pickles	/1		
*Prunus cerasus* L.			ķirši, вишня	C	leaves	fresh	condiment for pickles	1/1	2/1	6
					twigs	fresh	recreational tea	1		
*Rosa* spp.		DLGA050, DLGA068d	šopovņiks, mežrozītes, шипoвник, mežrozīte, рoза	W	flower petals	dried	recreational tea			1
					fruits	cooked	jam	1	1	
							syrup		1	
						dried	recreational tea	1		1
						fresh	snacks	1	1	1
*Rubus chamaemorus* L.			lācenes	W	fruits	fresh	snacks	2		
						frozen	dessert	1		
*Rubus idaeus* L.		DLGA012, DLGA033	малина, meža avenes, avenes, aveņis *, maļina, dārza avenes, малинник	W	fruits	cooked	compote	2	4	/1
							drink			/1
							jam	6	8	6/2
							juice		2	1
						fermented	wine	1	1	1
						fresh	snacks	3	2/1	3
						frozen	dessert		5	1
					leaves, twigs	fresh, dried	recreational tea	3	7/1	7
*Rubus nessensis* Hall			ежевика, kazene	W	fruits	cooked	jam		1	1
					leaves	dried	recreational tea	1		
*Sorbus aucuparia* L.		DLGA028	pīlādži, рябина	W	fruits	cooked	jam	2		
						fresh	snacks	1	1	/1
						dried	recreational tea		1	
*Populus spp.*, including *Populus balsamifera* L. and other cultivated species	Salicaceae		topoļi *^	W	buds	dried	recreational tea	/1		
*Salix* spp.			pūpoli	W	wood	burned	smoking meat	1		
*Acer platanoides* L.	Sapindaceae		kļava, kļavas, клён, кленoвый	W	leaves	fresh	under bread	/2	/1	/2
					sap	fresh, frozen	drink	12		5/1
*Aesculus hippocastanum* L.			kastaņi	W	nuts (conkers)	dried	recreational beverage	/1		
*Bergenia crassifolia* (L.) Fritsch	Saxifragaceae		бадан	C	leaves	dried brown leaves, collected in spring	recreational tea		1	
*Verbascum thapsus* L.	Scrophulariaceae	DLGA014	deviņvīru spēks	W	flowers	tea	drink	/1		
*Urtica* spp. including *Urtica dioica* L., *Urtica urens* L.,	Urticaceae		крапива, стрикава, nātres, nuotras *, nātras	W	aerial parts	cooked	cutlets		1	
							dumplings		1	
							soup	5/1	4/2	4/3
						dried	recreational tea		/1	
						scaled	salad	2	4	1/1

Abbreviations: C—cultivated, W—wild,; LG—Latgalians, MX—Mixed (non-Old Believers), OB—Old-Believers; x/—refers to current use; /x—refers to additional uses from the past; @—cultivated plant used in an unusual manner; ©—grows wild but often the cultivated plant is used; ^^—Latgalian dialect origin borrowed by Latvian speakers; ^—Latvian-speaking respondent providing Russian plant name, *^—Russian language origin borrowed by Latgalians speakers, *—Latgalian name.

**Table 3:** Quantitative comparison of taxa and uses between and within the groups.

Groups	Total Number of Taxa (Families) for Current Food Uses	Number of Taxa for Current Food Uses	Total Number of Taxa for Food Uses Past Uses Included (Families)	Number of Taxa for Food Uses (Past Uses Included)	Average Number of Taxa per Interviewee (Current Uses Only)	Min/Max Value of Taxa by Interviewees	Number of DUR—all Current Uses	Average Number of Current Food DUR per Interviewee	Min/Max Value of DUR by Interviewees
Old Believers (OB, *n* * = 19)	64 (34)	35	76 (38)	40	8 ± 4	3/14	197	10 ± 5	3/20
Latgalians (LG, *n* = 28)		52		63	9 ± 5	1/23	288	11 ± 7	1/34
Mixed (MIX, *n* = 26)		46		53	8 ± 5	1/18	263	11 ± 6	1/22
Mean number		44		52	8		249	10	
SD		8		11	1		47	1	

* *n* = total number of interviewees. Only interviewees reporting plants for food uses were considered for analysis.

## Data Availability

The data that support the findings of this study are available from the corresponding author upon reasonable request.
